# Case report: Identification of acute promyelocytic leukemia during osimertinib resistance followed by granulocyte colony-stimulating factor and pembrolizumab

**DOI:** 10.3389/fonc.2022.1032225

**Published:** 2023-01-13

**Authors:** Huohuan Tian, Linhui Yang, Wang Hou, Yu Wu, Yang Dai, Jiang Yu, Dan Liu

**Affiliations:** ^1^Department of Respiratory and Critical Care Medicine, West China Hospital, Sichuan University, Chengdu, Sichuan, China; ^2^Department of Hematology, West China Hospital, Sichuan University, Chengdu, Sichuan, China; ^3^Department of Laboratory Medicine, West China Hospital, Sichuan University, Chengdu, Sichuan, China

**Keywords:** therapy-related acute promyelocytic leukemia, osimertinib, granulocyte colony-stimulating factor, pembrolizumab, lung cancer

## Abstract

**Background:**

The occurrence of acute promyelocytic leukemia (APL) during the management of lung cancer is rare and life-threatening. It was mainly reported to be secondary to chemoradiotherapy. A few studies reported an increased incidence of therapy-related acute promyelocytic leukemia (t-APL) after gefitinib became available.

**Case presentation:**

We reported a patient who developed thrombocytopenia after receiving oral osimertinib in combination with intensity-modulated radiotherapy (IMRT). For half a year, she had an unrecoverable low platelet count, which progressed to concomitant leukopenia and the transient appearance of orthochromatic normoblasts in the peripheral blood test, indicating a dormant myeloid disorder. Due to simultaneous resistance to epidermal growth factor receptor (EGFR) tyrosine kinase inhibitors (TKI), pembrolizumab and granulocyte colony-stimulating factor (G-CSF) were administered, revealing prominent signs of hematological malignancy in a peripheral blood test that was later identified as t-APL.

**Conclusion:**

In general, patients undergoing EGFR-TKI combined with local radiotherapy should be concerned about their hematological assessment. If patients exhibit unrecoverable abnormalities in routine blood tests, a secondary nonsolid malignancy other than myelosuppression should be considered, and further lung cancer treatment should be discontinued.

## Introduction

Therapy-related acute myeloid leukemia (t-AML) secondary to the administration of chemotherapy and radiotherapy has been considered an exceptional and serious complication with an antecedent malignancy (1). As a fatal subtype of AML, acute promyelocytic leukemia (APL) is commonly characterized by a balanced chromosomal translocation between chromosomes 15 and 17 t-(15;17) (q24; q21), which leads to promyelocytic leukemia (PML)-retinoic acid receptor-α (RARα) rearrangement (2). Comparatively, t-APL may be favored to harbor additional cytogenetic abnormalities, commonly occurring in chromosomes 5, 7, and 17 (3, 4).

According to a systematic review, therapy-related APL following breast cancer is frequent in clinical practice (5). There are scattered reports of APL secondary to lung cancer and corresponding treatment.

Chemotherapy, radiotherapy, or both for prior neoplasms are demonstrated contributors to the development of t-APL (4, 6, 7). Current therapeutic strategies for lung cancer have flourished due to the emergence of targeted agents, immunotherapy, and the transition from conventional radiotherapy to intensity-modulated radiotherapy (IMRT). However, there is scarce evidence regarding APL in these novel therapies (8, 9).

Herein, we report a case of t-APL with advanced non-small cell lung cancer (NSCLC) after targeted therapy and IMRT followed by granulocyte colony-stimulating factor (G-CSF) and immune checkpoint inhibitor (ICI) treatment. Since t-APL shows a similar remission rate to *de novo* APL (80%) but a high risk of life-threatening coagulopathy (5, 10), we delineated the details so that clinicians may timely notice and treat the complication during the management of lung cancer.

## Case report

A 58-year-old woman was diagnosed with lung adenocarcinoma with an EGFR-L858R mutation and thoracic vertebral (T5 and T11) invasion in January 2021. Osimertinib was started in February 2021 at a dose of 80 mg/day. The patient received IMRT at the T5 (3,000 cGy 10 times, 2,100 cGy 7 times) and T11 (3,000 cGy 10 times, 2,100 cGy 7 times) from 3 to 17 June 2021 and 24 June to 5 July 2021, respectively. In June 2021, the patient complained of subtle ostealgia, and her laboratory tests revealed mild thrombocytopenia. While continuing osimertinib, the patient was followed up every 2 months until she achieved partial remission (PR). During that time, her platelet count was consistently 50–90 × 10^9^/L (**Table 1**), with no signs of hemorrhage.

**Table 1:** Peripheral blood tests during osimertinib and adjuvant IMRT.

	2,021.6.10	2,021.7.1	2,021.8.5	2,021.10.25	2,021.12.21	2,022.1.24	2,022.3.21	2,022.4.30
Hb (g/L)	130	129	112	125	127	128	119	116
WBC (10^9^/L)	5.5	3.97	3.97	4.14	3.73	3.84	3.08	1.9
PLT (10^9^/L)	83	58	55	66	81	86	68	37
ON (/100 cells)	0	0	0	0	0	0	0	1

Hb, hemoglobin; WBC, white blood cell; PLT, platelet; ON, orthochromatic normoblast; IMRT was started on 3 June 2021.

**Figure 1:**
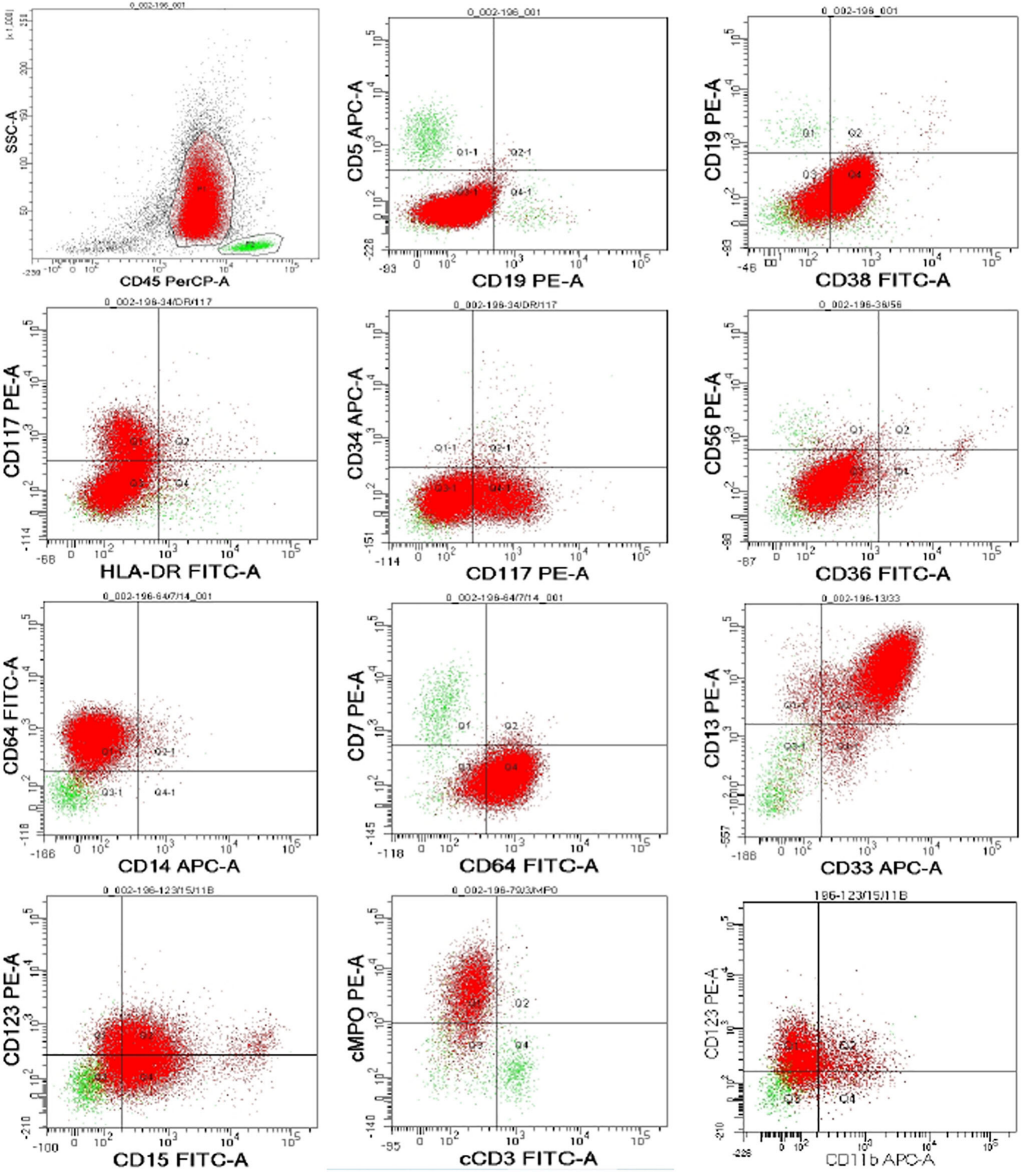
Bone marrow flow cytometry.

**Figure 2:**
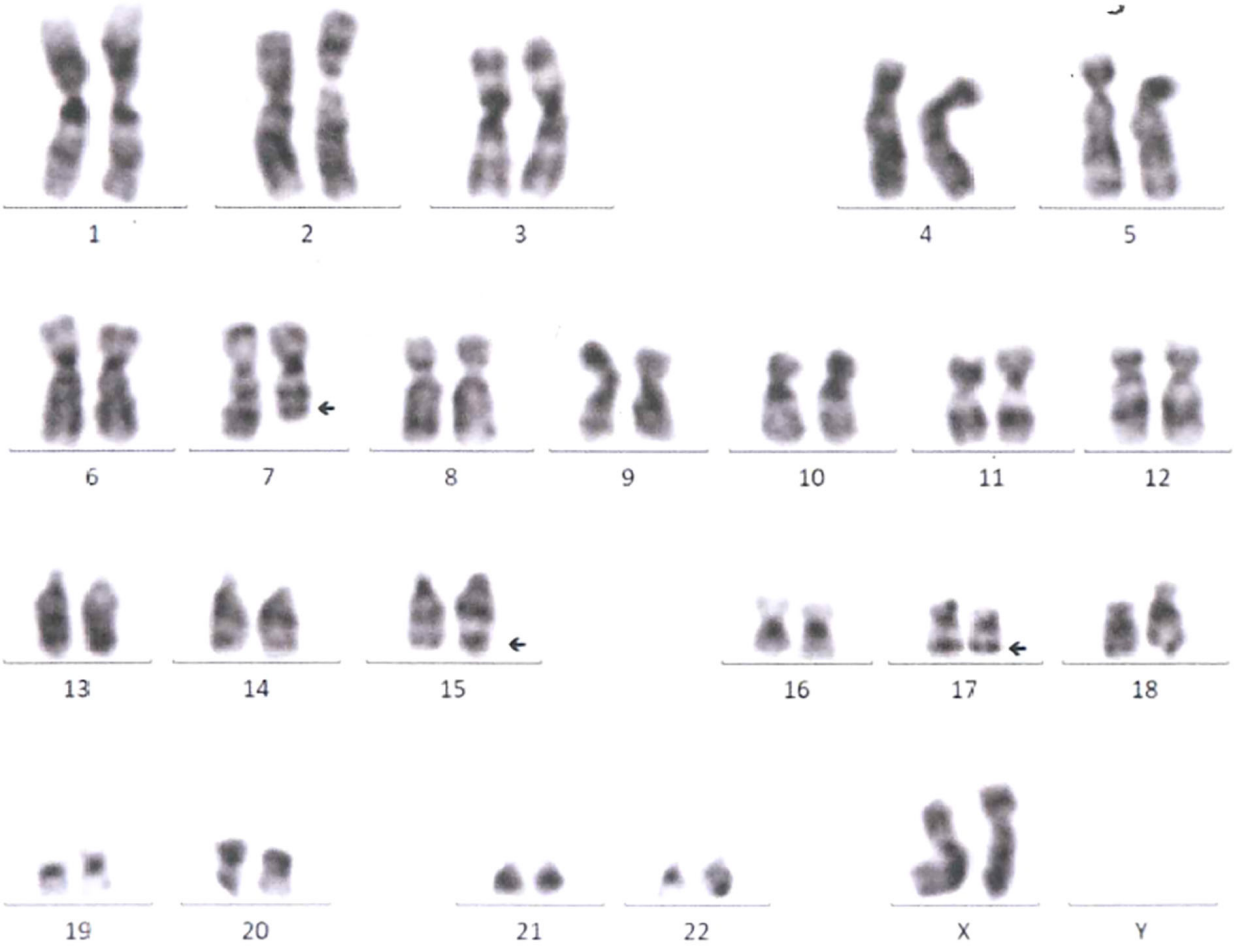
Marrow chromosomal analysis.

In March 2022, the patient reported a mildly aggravated ostealgia. A computed tomography (CT) scan in March 2022 revealed metastasis in the left humerus and sternum. Considering EGFR-TKI resistance, the clinician performed a tissue rebiopsy after withdrawing osimertinib. Peripheral blood test upon admission displayed thrombocytopenia, leukopenia, and orthochromatic normoblast appearance (**Table 1**), and coagulation parameters are shown in **Supplementary Table S1**. The pathological result of the rebiopsy is shown in **Supplementary Table S2**. Because spontaneous thrombocytopenia remission was predicted on 7 June 2022 (**Table 2**), immunotherapy combined with platinum-based chemotherapy was recommended. On the day when pembrolizumab at 200 mg/dl was administered, the patient experienced severe ostealgia all night. After receiving G-CSF and interleukin (IL)-11, myeloblasts containing Auer’s body appeared in her peripheral blood. Considering the possibility of APL, we monitored her coagulation parameters (**Supplementary Table S1**). A bone marrow aspiration was performed after stopping G-CSF and IL-11 treatment for 3 days. The bone marrow cytology revealed 68% promyelocytes, indicating t-APL (**Supplementary Figure S1**). Marrow flow cytometry (FCM) confirmed the finding (**Figure 1**). The identification of a rare PML/RARα (Bcr 3) fusion gene suggested acute myeloid rearrangement. There was no mutation in any common AML prognostic gene, including FLT3, dupMLL, IDH1, IDH2, NPM1, KIT, NRAS, CEBPA, DNMT3A, PHF6, TET2, ASXL1, RUNX1, TP53, and WT1. Chromosome karyotypic analysis showed 46, XX, add (7, 11), t (15;17) (q24; q21) [6]/46, XX [14] (**Figure 2**). Subsequently, the patient underwent all-trans retinoic acid and arsenic acid induction therapy. During the induction therapy, fever, hypotension, and pleural effusion occurred. Dexamethasone and vasopressor agents were also administered. She was successfully cured eventually. In September 2022, she received chemotherapy for lung cancer after documenting the complete remission of APL. She is now being followed up by telephone every month.

**Table 2:** Peripheral blood tests during usage of G-CSF and pembrolizumab.

	2,022.5.1	2,022.5.3	2,022.5.5	2,022.5.9	2,022.6.7	2,022.6.10	2,022.6.13
Hb (g/L)	103	96	104	96	96	89	94
PLT (10^9^/L)	36	35	42	63	97	49	49
WBC (10^9^/L)	4.64	2.77	2.91	2.15	1.49	4.13	4.74
ON (/100 cells)	0	0	0	1	0	1	0
Myeloblast (10^9^/L)	0	0	0	0	0	0	2
Promyelocytes (%)	0	0	0	0	0	0	1
G-CSF	+	−	+	+	−	+	+

Hb, hemoglobin; WBC, white blood cell; PLT, platelet; ON, orthochromatic normoblast; G-CSF, granulocyte colony-stimulating factor; “+” G-CSF was injected; “−” G-CSF was not used.

The patient had no history of any hematological disease that could lead to acute leukemia, such as lymphoma, myelodysplastic syndromes (MDS), multiple myeloma, and so on. She denied any hematological disease history in her family members.

## Discussion

EGFR-TKI ± radiotherapy is the first-line treatment for advanced NSCLC patients with EGFR mutation and oligometastasis (12). Studies demonstrated that radiation was the inciting factor for myeloid neoplasms, including all AML categories and MDS (5, 7, 13). Lung cancer treated with radiation showed an increased relative risk of AML/MDS over the next 1–12 years (14). Ionizing radiation may prompt reactive oxygen species, causing DNA double-strand breaks (15). However, the radiotherapy modality research involved mainly encompassed external beam therapy (EBRT) and brachytherapy. The use of IMRT increased in the past few years because of its superior safety and fewer side effects. Modulated radiation beams and sculpted radiation doses ensure precise coherence with geometric targets and enhanced therapeutic effects (16). A study revealed that secondary MDS/AML could be provoked by IMRT in prostate cancer patients (17). Hematological toxicity, such as thrombocytopenia, was also observed in malignant pleural mesothelioma patients treated with IMRT (18). However, it has not been reported as a factor for hematological malignancy or toxicity in lung cancer patients.

Patients with radiation-induced secondary malignancies tend to experience a long latency period (19, 20). Paradoxically, the intervals between initial radiotherapy and our patient’s thrombocytopenia and t-APL diagnosis were 7 days and 12 months, respectively. In 2006, Keitaro et al. observed a clustered incidence of acute promyelocytic leukemia in NSCLC cases treated with first-generation EGFR-TKI gefitinib (8, 21). Notably, all the observed cases reported cytopenia, especially thrombocytopenia in the beginning. The authors suggested that the t-APL inducibility of gefitinib should be further elucidated. In 2016, clinicians from Japan found a chronic myelomonocytic leukemia blast crisis when synchronous lung adenocarcinoma was treated by EGFR-TKI (22). Another study in 2021 reported a case of thrombocytopenia with immature cells in the peripheral blood during receiving erlotinib, which turned out to be t-AML (11). To date, no report has identified hematological malignancies while the patient was receiving osimertinib treatment. We identified transient orthochromatic normoblasts in our patient’s peripheral blood on 30 April 2022, during osimertinib treatment in our case. Furthermore, subsequent G-CSF and IL-11 after TKI cessation did not ameliorate her thrombocytopenia and emerging leukopenia from 30 April 2022 to 9 May 2022 (**Table 2**), signifying a hematopoiesis disorder such as MDS and acute myeloid leukemia. Hence, we speculated that continuous usage of third-generation EGFR-TKI osimertinib may hasten the secondary APL. Regrettably, bone marrow aspiration was not performed when cytopenia occurred during continuous EGFR-TKI and regional radiotherapy treatment.

T-APL was not confirmed until the administration of a second-line therapy comprising pembrolizumab and chemotherapy. G-CSF is a hematopoietic glycoprotein produced by monocytes, macrophages, fibroblasts, and endothelial cells (23). It is used to accelerate neutrophil recovery after chemotherapy by regulating cell cycle activation, proliferation, and terminal maturation (23). Researchers revealed that congenital neutropenia patients on G-CSF are more susceptible to developing MDS/AML over time (24). Granulocyte colony-stimulating factor has also been shown to be a risk factor for AML/MDS in breast and lung cancer populations (25). Promyelocytes containing Auer’s body indeed emerged in our patient’s peripheral blood after G-CSF usage. Nevertheless, progressive thrombocytopenia, leukopenia, and orthochromatic normoblast were virtually presented in her peripheral blood before G-CSF usage, when she was still undergoing osimertinib treatment. Considering that t-APL typically occurs without the prodromal, preleukemic, and myelodysplastic phases (5), we speculated a likelihood of t-APL existence prior to the use of G-CSF.

Immunotherapy plays a critical role in NSCLC. PD-1-binding pembrolizumab disrupted its anchoring to PD-L1, impeding the inhibition of T cells and mounting its recognition to tumor cells (26). There is only one case report to date about t-APL involving the history of pembrolizumab treatment (9). According to our observation, the intravenous infusion of pembrolizumab aggravated the patient’s chronic ostealgia acutely. The association between pembrolizumab and t-APL remained occult. Of interest, the refractory leukopenia was alleviated but the thrombocytopenia was exacerbated following pembrolizumab and G-CSF (**Table 2**). This phenomenon resembled Paola’s study, which found that G-CSF injection worsened anemia in breast cancer patients receiving chemotherapy (27), and it is hypothesized that granulopoietic lineages competed with erythropoietic lineages for differentiating hematopoietic cell stems.

Altogether, our patient had been exposed to a series of predisposing factors to t-APL, making it difficult to ascertain that the third-generation EGFR-TKI osimertinib was the sole and determining cause of the complication. However, it is reasonable to conclude that the myeloid disease developed during IMRT plus continuous osimertinib. Further evaluation based on a larger sample size is warranted. Regardless, a widespread matter of osimertinib resistance gives rise to novel therapy exploration (28). KEYNOTE-789 is an ongoing trial assessing platinum-based chemotherapy combined with pembrolizumab for this entity. EGFR-TKI, radiation, G-CSF, and chemotherapy agents are all widely used, increasing the risk of a secondary hematological disease and even malignancy. Given the positive response of t-APL to all-trans retinoic acid therapy, a key problem now is determining whether we can identify those nearly asymptomatic patients early and withhold APL-facilitating interventions. Thus, after the patients achieved hematological remission, should the treatment strategy for lung cancer be persisted?

## Conclusion

We first reported the identification of t-APL when the patient acquired resistance to osimertinib. Chemotherapy, radiotherapy, or both have been demonstrated to be risk factors for t-APL. Previous reports and our case supported positive concern for such complications, given the widespread usage of EGFR TKI and resistance to targeted therapy

## Data availability statement

The original contributions presented in the study are included in the article/**Supplementary Material**. Further inquiries can be directed to the corresponding author.

## Ethics statement

The studies involving human participants were reviewed and approved by Sichuan University Ethics Review Board. The patients/participants provided their written informed consent to participate in this study. Written informed consent was obtained from the individual(s) for the publication of any potentially identifiable images or data included in this article.

## Author contributions

HT, LY, and WH: writing—original draft. YW, YD, and JY: writing original draft preparation. DL: administrative support and supervision. All the authors contributed to the article and approved the submitted version.

## Funding

This work was supported by National Natural Science Foundation of China (Grant Number 82173182).
