# Supplementary material for: Case report: Identification of acute promyelocytic leukemia during osimertinib resistance followed by granulocyte colony-stimulating factor and pembrolizumab
Source: Front Oncol. 2023 Jan 13;12:1032225. doi: 10.3389/fonc.2022.1032225 (PMC9880289; doi:10.3389/fonc.2022.1032225)
Supplement: Supplementary file 1 [file Table_1.docx]

Table S1 Patient’s coagulation parameters.

|  | 2022.4.30 | 2022.6.7 | 2022.6.16 | 2022.6.20 | 2022.6.23 | 2022.6.28 | 2022.6.30 |
| --- | --- | --- | --- | --- | --- | --- | --- |
| PT (s) | 10.3 | 10.5 | 11.2 | 10.8 | 11.0 | 10.7 | 9.6 |
| APTT (s) | 25.6 | 22.5 | 27.1 | 24.8 | 26.8 | 31.0 | 27.0 |
| Fib (g/L) | 3.80 | 3.07 | 4.20 | 3.55 | 4.38 | 2.74 | 2.22 |
| D-dimer (mg/I FEU) | 1.65 | 8.18 | 11.53 | 12.02 | 9.47 | 4.09 | 2.82 |
| AT-III (%) | 100.8 | 103.1 | 76.2 | 85.8 | 93.5 | 74.2 | 84.2 |

PT, Prothrombin time; APTT, Activated partial thromboplastin time; Fib, Fibrinogen; AT-III, Antithrombin-III.

Table S2 Pathological results of re-biopsy.

| Gene | Variation | Mutant type | Tissue abundance |
| --- | --- | --- | --- |
| EGFR | p.L858R nonsense mutations in exon 21 | c.2573T>G (p.L858R) | 37.7% |
| TP53  TSC2  EGFR  EPHA3  PIK3R2  POLE  RAD50 | p.Q317 nonsense mutations in exon 9  c.3285-1G>A spliced mutation in intron 28  p. L62R missense mutation in exon 2  p.R868T missense mutation in exon 15  p.N403I missense mutation in exon 10  p. S233P missense mutation in exon 7  p.I4M missense mutation in 1 exon | c.949C>T (p.Q317)  c.3285-1G>A  c.185T>G (p. L62R)  c.2603G>C (p.R868T)  c.1208A>T (p.N403I)  c.607T>C (p. S233P)  c.12C>G (p. I4M) | 38.8%  12.8%  43.4%  0.8%  26.2%  29.2%  5.7% |
