# Supplementary figures and images for: Case report: Identification of acute promyelocytic leukemia during osimertinib resistance followed by granulocyte colony-stimulating factor and pembrolizumab
Source: Front Oncol. 2023 Jan 13;12:1032225. doi: 10.3389/fonc.2022.1032225 (PMC9880289; doi:10.3389/fonc.2022.1032225)

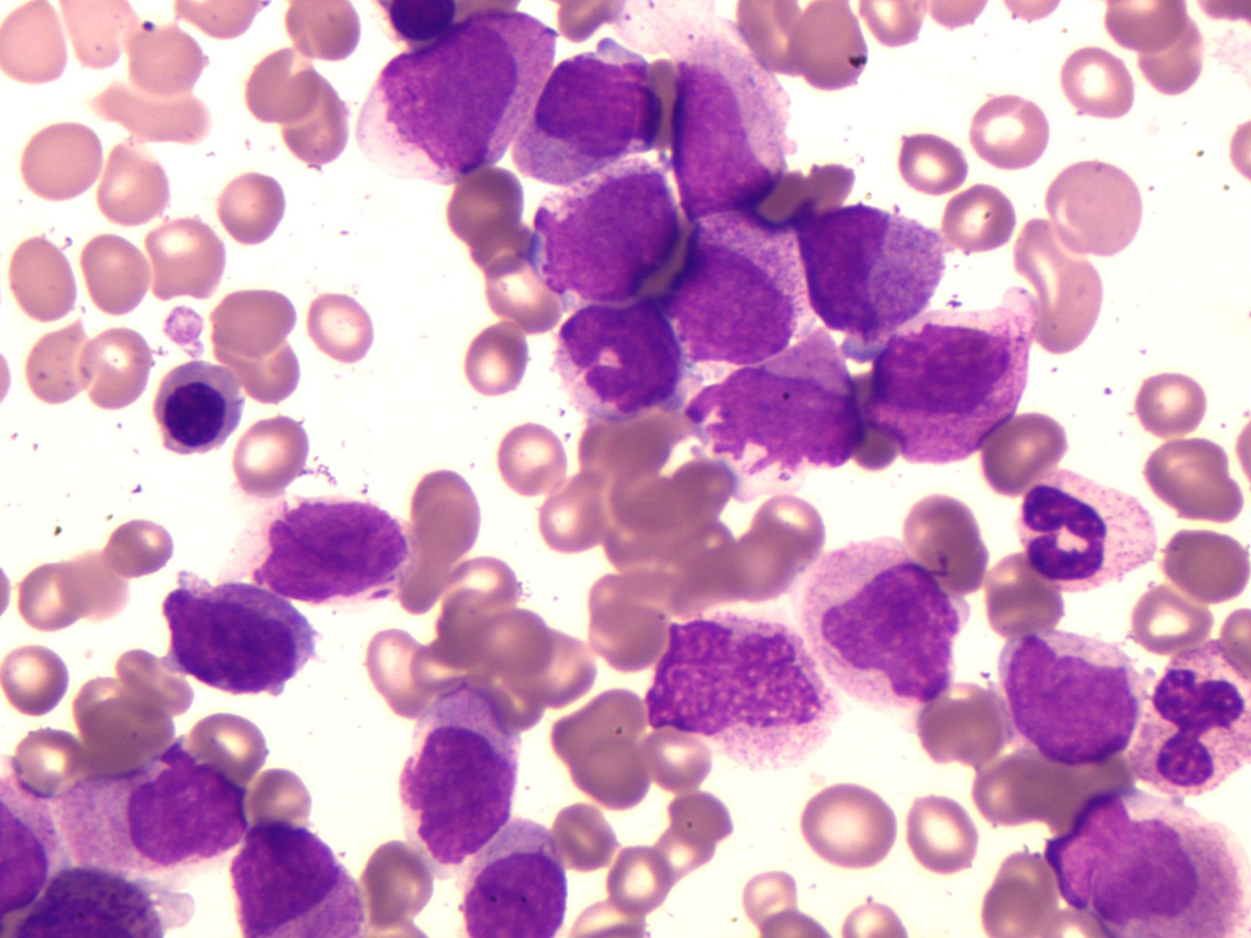

Supplement: Supplementary file 2 [file Image_1.tif]
